# Narrative-driven virtual reality serious game to support type 1 diabetes self-management in children

**DOI:** 10.1038/s41598-025-30114-1

**Published:** 2025-12-03

**Authors:** Hanfei Gu, Nadia Diyana Binti Mohd Muhaiyuddin, Nassiriah Binti Shaari

**Affiliations:** School of Multimedia Technology and Communication, Universiti Utara Malaysia, 06010 Sintok, Bukit Kayu Hitam, Kedah Malaysia

**Keywords:** Type 1 diabetes (T1D), Children, Self-management, Virtual reality (VR), Serious games, Human–computer interaction (HCI), Participatory design, Digital health, Health care, Psychology, Psychology

## Abstract

Supporting type 1 diabetes (T1D) self-management in young children remains a major challenge. Traditional education often fails to sustain attention or meet developmental needs. This study introduces *Tangbao Superman Transformation*, a narrative-driven virtual reality (VR) serious game developed through participatory Human–Computer Interaction (HCI) methods, to enhance pediatric T1D self-management. A four-week single-group pre–post feasibility study was conducted with 54 children aged 4–9 years recruited from four pediatric hospitals in eastern China. The intervention translated the Chinese Diabetes Society’s seven-domain (CDS-7) framework into VR modules co-designed with children, parents, and clinicians. Quantitative measures included the Self-Management Scale for T1D Children (SMS-T1DC), the System Usability Scale for Children (SUS-C), and an Engagement and Motivation Questionnaire (EMQ). Attendance logs and field notes documented adherence and safety, while semi-structured interviews with children, parents, and providers provided qualitative insights. Children showed significant improvements across all seven self-management domains (*p* < 0.001; Cohen’s d = 0.78–1.27). Usability was rated as excellent (SUS-C = 86/100), engagement was high (95% session completion), and qualitative findings highlighted reduced resistance, increased confidence, and application of strategies in daily routines. This feasibility study provides preliminary evidence that participatory, narrative-driven VR interventions can enhance pediatric T1D self-management. However, the absence of a control group, short intervention duration, lack of biomedical endpoints, and sample homogeneity limit generalizability. Future trials should incorporate active comparators (traditional teaching or mobile apps), biomedical outcomes, multi-center recruitment, and long-term validation to establish incremental efficacy and scalability.

## Introduction

Type 1 diabetes (T1D) is among the most common chronic autoimmune diseases in childhood, and its incidence continues to rise globally in both developed and developing countries^1,2^. Effective management extends beyond clinical treatment and depends heavily on children and their families consistently performing daily self-care activities such as insulin administration, blood glucose monitoring, dietary regulation, physical activity, and psychosocial adjustment^3,4^. Yet, establishing sustainable routines remains especially challenging for younger children, who often struggle with abstract health concepts, fluctuating motivation, and limited capacity for long-term planning^5,6^.

While families and schools provide ongoing education, meaningful behavioral change is difficult to achieve without interventions that are developmentally appropriate and intrinsically engaging^6^. Recent advances in digital health—particularly serious games and immersive virtual reality (VR)—have shown potential to enhance pediatric self-management by embedding learning into play and sustaining attention more effectively than traditional didactic methods^7–9^. VR can provide safe, repeatable, and personalized practice environments, turning routine education into salient and enjoyable experiences^10^. However, most VR health interventions have been developed for adolescents or adults, leaving a critical gap in evidence-based designs tailored for younger children^11^.

Developing child-centered digital health solutions requires more than clinical accuracy. Researchers in Human–Computer Interaction (HCI) must ensure that interventions are engaging, developmentally aligned, culturally relevant, and ethically sound^12,13^. This calls for participatory design, usability testing, inclusive strategies, and rigorous ethical safeguards integrated across all phases of development^14,15^. Yet, few studies systematically apply HCI frameworks in both design and evaluation of VR interventions for T1D, with existing work often focusing narrowly on clinical or educational outcomes while neglecting lived user experiences, levels of child participation, and real-world accessibility^16–18^.

To address these gaps, the present study aims to design and evaluate *Tangbao Superman Transformation*, a narrative-driven virtual reality (VR) serious game for children aged 4–9 years to support type 1 diabetes (T1D) self-management. Guided by contemporary Human–Computer Interaction (HCI) principles, the project actively involved children, parents, and healthcare providers throughout the participatory design and evaluation process. The study pursues three specific objectives: (1) to advance participatory design methodologies for VR health interventions with young children; (2) to operationalize the Chinese Diabetes Society’s seven-domain self-management framework (CDS-7)^19^ into engaging and developmentally appropriate VR mechanics; and (3) to empirically evaluate the feasibility, usability, and ethical soundness of the intervention in real-world pediatric contexts.

As a multidisciplinary initiative, this study seeks to contribute theoretical and practical insights to the fields of educational technology, digital health, and child–computer interaction. It also aims to extend HCI scholarship by demonstrating how child-centered methodologies can inform the design of digital tools for chronic disease self-management^8^. Building on this foundation, subsequent research will focus on scaling the approach to larger and more diverse populations, incorporating long-term follow-up, and integrating biomedical outcomes such as HbA1c and continuous glucose monitoring (CGM) to assess sustained clinical impact.

## Related work

### Child-centered and participatory design in HCI

There is broad consensus in Human–Computer Interaction (HCI) that traditional top-down development approaches are inadequate for pediatric digital health^20^. Research in child–computer interaction demonstrates that involving children as active co-creators—rather than passive recipients—enhances usability, engagement, and ecological validity^21,22^. Frameworks such as *Cooperative Inquiry* and *Participatory Action Research* illustrate how children’s authentic perspectives can reveal design needs that adult-centric approaches often overlook^23,24^.

In this project, multiple rounds of co-creation workshops and family interviews ensured alignment with children’s cognitive, emotional, and motivational needs, while also surfacing practical barriers. Literature confirms that genuine behavioral change and sustained engagement occur only when children’s lived experiences are embedded into the design process^17,22^, making participatory design a cornerstone of HCI-driven pediatric health innovation.

### Serious games, VR, and health behavior change

Serious games and immersive virtual reality (VR) are increasingly recognized as promising tools for pediatric health promotion^25^. By embedding complex medical concepts into interactive experiences, these technologies can enhance knowledge acquisition, self-efficacy, and intrinsic motivation^26,27^. For example, Ludlow et al.^28^ demonstrated that VR interventions adapted to developmental stages improved learning outcomes and treatment adherence compared with traditional approaches.

Recent reviews emphasize that effective interventions must tightly integrate validated medical knowledge with immersive game mechanics^27,29^. For children with type 1 diabetes (T1D), this integration enables safe and repeated practice of essential self-management skills^30^. However, most studies measure only short-term knowledge or motivation, lack control groups, and rarely incorporate clinical biomarkers such as HbA1c or continuous glucose monitoring (CGM). Developmental differences among younger age groups are also underexplored^25^.

The Chinese Diabetes Society’s seven-domain model (CDS-7) offers a comprehensive framework for pediatric self-management, spanning knowledge, diet, decision-making, glucose monitoring, insulin use, physical activity, and psychosocial well-being^19^. Despite its relevance, few digital health interventions systematically map VR mechanics to CDS-7 domains—an important gap that this study directly addresses.

## Research gap

Although international research has begun to validate serious games and VR in healthcare, few studies systematically apply HCI frameworks or evaluate interventions in real-world pediatric contexts. Existing work typically focuses on clinical or educational outcomes while overlooking key HCI dimensions such as child participation, cultural adaptability, and ethical safeguards^12,17^.

Critically, there remains a lack of integrated approaches that combine participatory design, narrative-driven VR gameplay, and evidence-based self-management frameworks. Few studies employ control groups, longitudinal designs, or clinical biomarkers, and cross-cultural validation is scarce^5,26^.

Evidence integrating the Chinese Diabetes Society’s seven-domain (CDS-7) framework with narrative-driven VR interventions for younger children with T1D is still limited. This study contributes by systematically operationalizing CDS-7 domains into VR game mechanics within a participatory HCI methodology and empirically validating the approach in pediatric care settings.

## Ethical considerations in child health technology

With growing adoption of digital health for children, issues of ethics, privacy, and inclusivity are central concerns in HCI. Ethical oversight and inclusive design are essential not only to protect individual participants but also to advance equity and justice in healthcare^12,18^. Yet prior interventions often under-address cultural inclusivity and the risk of negative reinforcement, which can increase anxiety or exclusion among children.

In this project, strict ethical guidelines approved by a medical ethics committee were followed. Parental consent and child assent were obtained using age-appropriate explanations and visual aids. Game content deliberately excluded elements that could induce shame, competition, or anxiety, instead emphasizing cooperation, growth, and positive reinforcement^13^. Representation across gender, ability, and cultural background was prioritized to maximize identification with the characters. This reflects a broader HCI commitment to safety, inclusivity, and equity in pediatric health technology^13^.

Establishing a comprehensive ethical framework is essential for digital health interventions involving children. From inception, the research team implemented a full-spectrum ethics management framework that prioritized child rights protection, data security, and psychological well-being. Drawing on the *Declaration of Helsinki*, CIOMS International Ethical Guidelines for Health-Related Research with Humans, and pediatric digital health recommendations^31,32^, four guiding principles were adopted: informed consent and assent, continuous process monitoring, dynamic risk management, and respect for voluntary withdrawal. These measures ensured adherence to beneficence, non-maleficence, autonomy, and justice throughout all phases of the study.

### Institutional approval and regulatory compliance

Formal approval was granted by the Medical Ethics Committee of Changzhou Children’s Hospital (Changzhou, Jiangsu Province, China). The study protocol was developed in consultation with pediatric endocrinologists, ethicists, legal experts, and child psychologists to ensure alignment with both national regulations and international standards for pediatric digital health research^33^. The workflow encompassed ethical review, privacy and security protocols, intervention testing, and transparent reporting, ensuring accountability and traceability at every stage^34^.

### Informed consent and child assent

Parents or legal guardians were fully informed of the study’s objectives, procedures, potential risks, and benefits, and written consent was obtained before enrollment. Child assent was sought through age-appropriate explanations, cartoon illustrations, and interactive discussions, enabling children to express willingness in a developmentally meaningful way. Families were clearly informed of their right to withdraw at any time, safeguarding autonomy and voluntariness.

### Data privacy, confidentiality, and security

Given the sensitivity of pediatric health data, multiple safeguards were implemented. These included data encryption, de-identification, and tiered access management, with raw data stored only on secure password-protected servers accessible to core research staff. Only anonymized and aggregated data were used in analysis, in accordance with Chinese data protection law and the EU’s General Data Protection Regulation (GDPR)^35^. All data were strictly limited to research purposes and never used for commercial applications.

### Psychological safety, well-being, and inclusivity

Psychological safety and inclusivity were prioritized in both design and implementation^36^. Reward and feedback systems emphasized cooperation, encouragement, and personal growth, avoiding punishment, humiliation, or competitive stress. Diverse character representation supported identification across gender, ability, and cultural backgrounds. All content and mechanics were reviewed by pediatric psychologists and child development specialists to ensure developmental appropriateness. Staff were trained to promptly address signs of fatigue, discomfort, or distress, and no adverse events were reported.

### Ongoing monitoring and post-study support

Professional staff continuously monitored children’s emotional and behavioral states in real time during design, gameplay, and evaluation. Sessions were adjusted or paused if concerns emerged. Following the intervention, children were invited to debriefing sessions to process experiences and reinforce positive outcomes. This principle of continuous and dynamic monitoring ensured participant welfare from ethical review through post-study follow-up, in line with international best practices for pediatric digital health research^31,32^.

## Methods

### Participatory design process

To ensure that the VR serious game closely reflected the real needs of children with type 1 diabetes (T1D), a participatory design methodology was systematically employed. Children (aged 4–9), their parents, diabetes educators, and pediatricians were invited to serve as co-design partners rather than passive informants, enabling the design to capture developmental, motivational, and clinical perspectives in a balanced way and ensuring that the intervention was safe, age-appropriate, and engaging.

Recruitment for design activities was conducted through hospitals and community centers using purposeful sampling to include variation in children’s ages and family backgrounds. Scenario-based prompts (e.g., “Who is your ideal diabetes warrior?”; “What happens when you encounter hypoglycemia?”) were used to stimulate imagination and encourage children to articulate expectations for characters, challenges, and rewards. Parents and healthcare providers joined parallel sessions to assess feasibility, clinical relevance, and safety concerns. The research team synthesized this input into iterative design changes spanning narrative arcs, interface metaphors, and interaction complexity.

The design proceeded through three iterative refinement cycles. After each cycle, supervised playtests were conducted in hospital playrooms, supported by observational field notes, video recordings, and debriefing interviews. Adjustments included simplifying voice prompts and UI density, enlarging interaction targets, and introducing optional rest breaks. When younger participants struggled with complex interactions, additional scaffolds were added; older children requested and received greater challenge levels. Pediatric endocrinologists and child psychologists reviewed each prototype iteration, providing expert input on developmental appropriateness, psychological safety, and clinical fidelity; recommendations were integrated to balance engagement with scientific rigor^14,15^.

### VR Prototype development

**Fig. 1:**
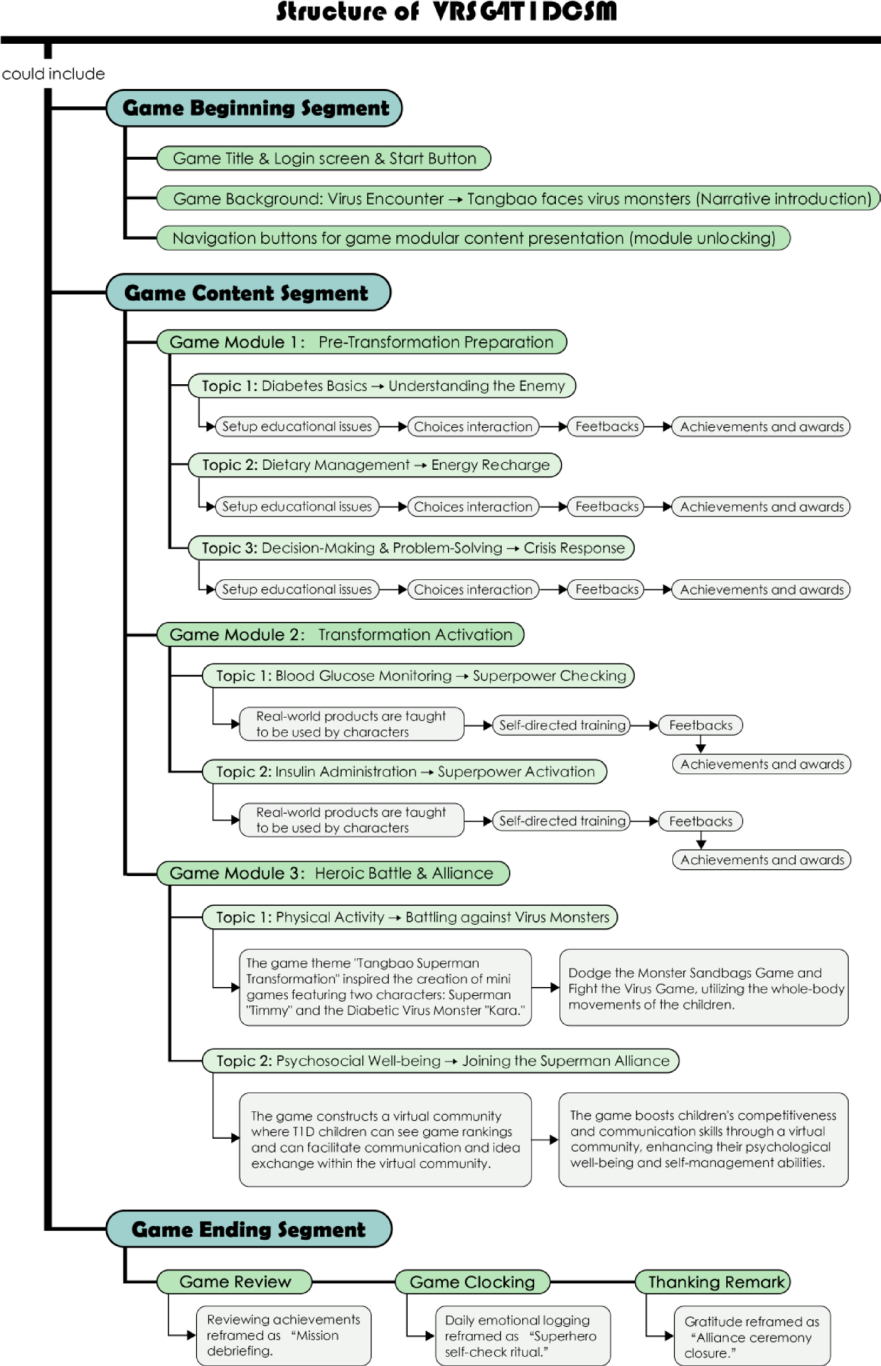
Overall Structure of the VRSG4T1DCSM Prototype *Note.* Arrows indicate progression across the three segments, with optional repetition loops for independent rehearsal.

*Tangbao Superman Transformation* was developed in Unity 2021 with a design philosophy emphasizing accessibility for early learners. Interfaces featured large interaction targets, progressive disclosure, and concise voice narration to minimize cognitive load. The prototype followed a three-segment structure (Fig. 1): (a) b**eginning**—login, narrative briefing, avatar customization; (b) **content**—seven training modules aligned with CDS-7; and (c) **ending**—progress review, achievement display, and closing animations reinforcing key health messages. Each module functioned as an independent, replayable learning unit (e.g., *Tangbao’s Origin Story* for disease basics; *Healthy Chef Challenge* for nutrition; *Super Sensing Mission* for glucose monitoring; *Friendship Garden* for psychosocial well-being).

### Mapping self-management content to game mechanics

The Chinese Diabetes Society’s seven-domain model (CDS-7) provided the pedagogical framework: disease knowledge, dietary management, decision-making in emergencies, blood glucose monitoring, insulin administration, physical activity, and psychosocial well-being. Each domain was operationalized as a distinct VR module with mechanics tailored to children’s developmental stages. Abstract medical concepts (e.g., glucose balance) were externalized into manipulable objects; emotionally challenging tasks (e.g., insulin administration) were scaffolded through playful metaphors and step-wise rehearsal. Correct strategies triggered positive reinforcement; errors elicited supportive guidance rather than punishment. The detailed mapping of these domains to specific game modules, mechanics, and learning outcomes is provided in Table 1.

**Table 1:** Mapping of CDS-7 Self-management domains to VR modules with narrative reframing.

Self-management domain	Game module (Theme)	Key game mechanics	Intended learning outcome	Narrative REFRAMING
Diabetes basics	Tangbao’s Origin Story	Interactive narrative, scaffolded Q&A	Understand foundational disease concepts	Classroom Q&A reframed as “Understanding the enemy virus.”
Dietary management	Healthy Chef Challenge	Food sorting, meal assembly mini-game	Identify healthy foods and appropriate meal timing	Kitchen scenario reframed as “Energy recharge for superpower.”
Decision-making & problem-solving	Emergency Adventure	Branching scenarios, consequence feedback	Choose appropriate responses to hypoglycemia	Hypoglycemia/social dilemmas reframed as “Crisis response with superhuman wisdom.”
Blood glucose monitoring	Super Sensing Mission	VR tool simulation, real-time alerts	Practice safe blood glucose monitoring	Monitoring reframed as “Superpower value check.”
Insulin administration	Energy Injection Quest	Stepwise guidance, procedural rehearsal	Learn correct insulin injection sequencing	Injection reframed as “Superpower activation ritual.”
Physical activity	Sports Hero	Motion tracking, avatar activity goals	Build daily exercise habits and self-monitoring	Jungle adventure reframed as “Battle against virus monsters.”
Psychosocial well-being	Friendship Garden	Social mini-games, achievement board	Foster peer support, confidence, and emotion regulation	Community hub reframed as “Joining the superhuman alliance.”

CDS-7 = Chinese Diabetes Society seven-domain model. Content mapping and age grading were reviewed by pediatric endocrinologists, certified diabetes educators, and child psychologists for accuracy, developmental fit, and ethical appropriateness.

**Fig. 2:**
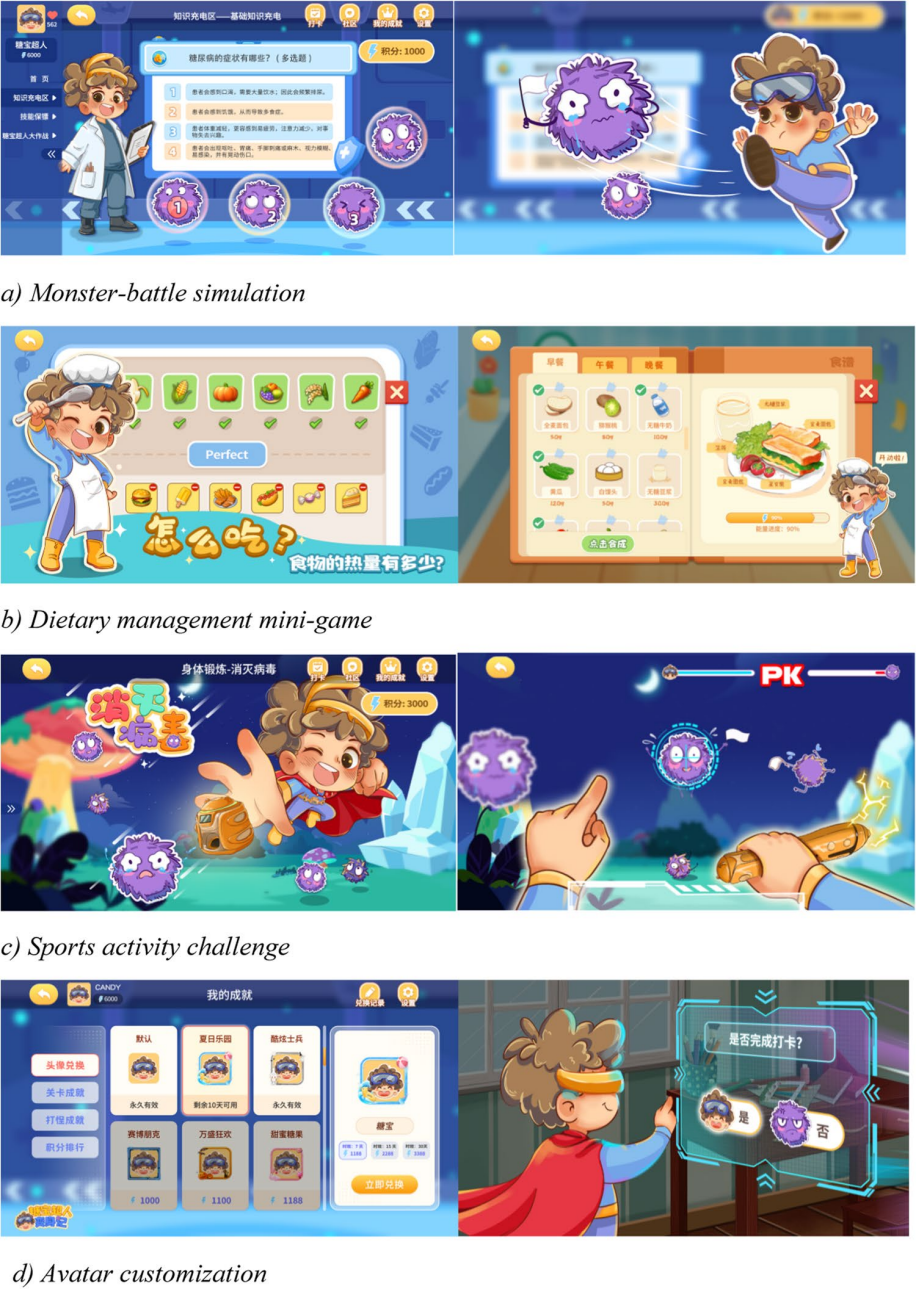

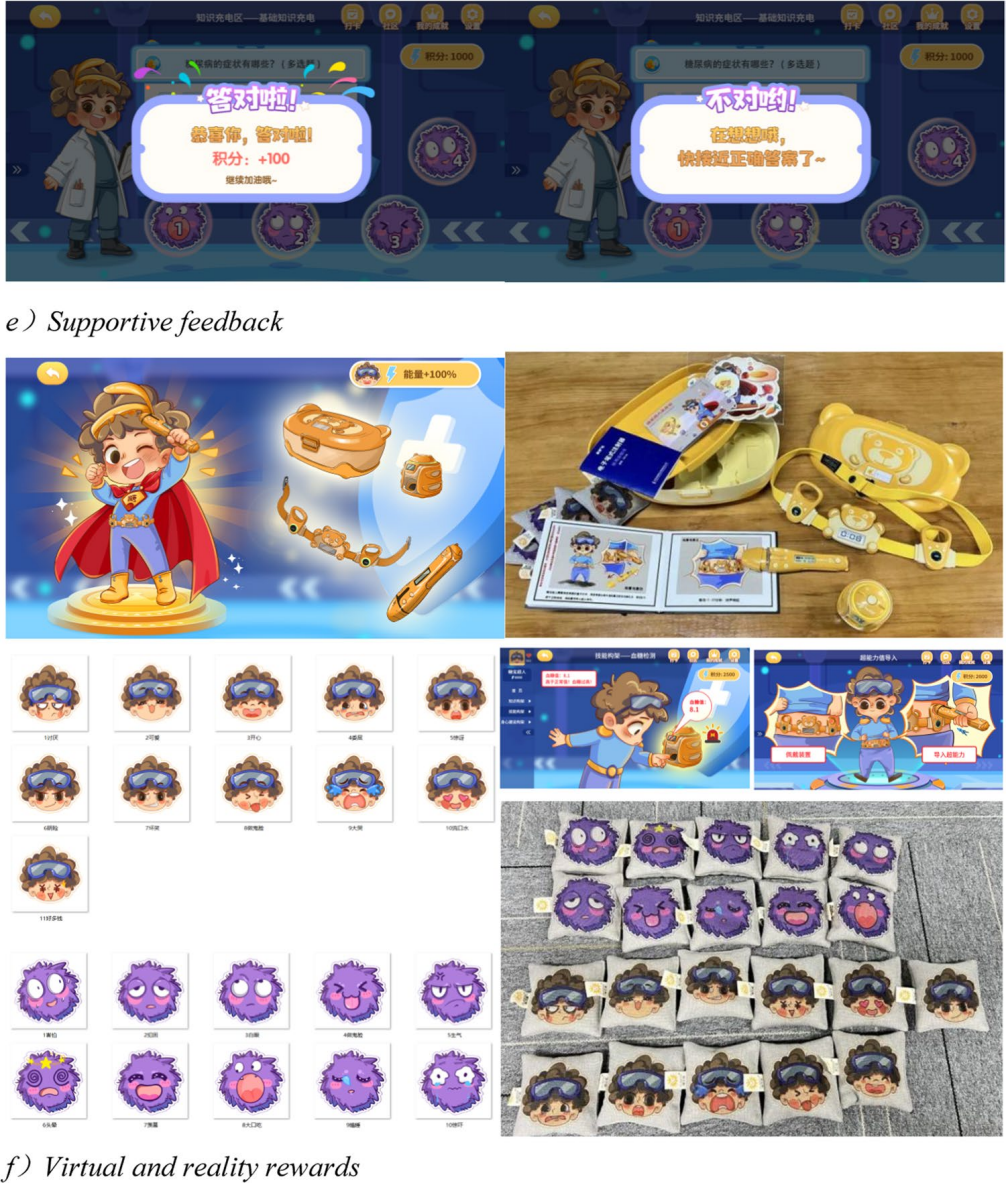
Representative Screenshots from the Tangbao Superman Transformation Prototype (**a**) Monster-battle simulation (metaphor for hyper/hypoglycemia management); (**b**) Healthy chef challenge (dietary management task); (**c**) Sports hero (physical activity with motion tracking); (**d**) Avatar customization (personalization and identity building); (**e**) Supportive feedback (encouraging dialogue and real-time guidance); (**f**) Virtual and reality rewards (achievement display, badges, and celebratory animations). All visual materials (photograph and VR screenshot) were created by the authors. *Note.* Screenshots correspond to the modules and mechanics listed in Table 1, demonstrating how abstract self-management routines were reframed into engaging, age-appropriate activities. Positive reinforcement mechanisms (supportive feedback, achievement boards, celebratory animations, and hybrid reward systems) were prioritized, while punitive features (e.g., failure screens) were deliberately excluded to prevent negative affect or social comparison. All modules and motivational elements were reviewed by pediatric endocrinologists, diabetes educators, and child psychologists to ensure developmental appropriateness, scalability, and ethical integrity.

Representative screenshots are provided in Fig. 2a–f. These examples illustrate how the prototype operationalized CDS-7 domains and integrated motivational design features. Scenes were optimized to minimize motion discomfort (e.g., smoothed transitions, reduced vection cues) and equipped with simplified controls to support younger players.

### Study design and quantitative evaluation

This single-group, pre–post mixed-methods study evaluated feasibility, usability, and preliminary effectiveness as proof-of-concept in a novel pediatric context^37^. While the absence of a control group limits causal inference, the design yields essential early-stage evidence on user experience, short-term learning, and potential behavioral change. Ethical approval was granted by the Medical Ethics Committee of Changzhou Children’s Hospital (Jiangsu Province, China), and procedures adhered to the Declaration of Helsinki. Written parental consent and age-appropriate child assent were obtained.

#### Participants and recruitment

Children aged 4–9 years with physician-confirmed T1D were recruited from four pediatric hospitals in eastern China. Recruitment posters and educator briefings were disseminated in outpatient clinics and community diabetes education programs. To minimize selection bias, clinicians and educators broadly distributed study information but did not directly recommend specific children. Inclusion criteria were the ability to participate in VR-based play and assessments; exclusion criteria included severe comorbidities (e.g., epilepsy, significant cognitive impairment) or lack of consent/assent.

#### Intervention protocol

The four-week protocol comprised: (a) baseline assessment; (b) supervised gameplay three times per week (20–30 min/session) in hospital playrooms or monitored community centers; and (c) post-test with repeated measures, usability surveys, and interviews. Trained facilitators oversaw sessions and offered optional breaks. Attendance logs and observational field notes documented adherence and safety.

#### Measures

##### Self-management scale for T1D children (SMS-T1DC)

The SMS-T1DC was developed as a core instrument to assess self-management behaviors in children across seven domains (knowledge, dietary management, decision-making/problem-solving, insulin administration, glucose monitoring, physical activity, psychosocial well-being). Items were adapted and integrated from multiple validated pediatric self-management questionnaires^38–43^, expanded to a pool of over 50, and refined through expert review and pilot testing with children. The final version comprised 36 items rated on a 5-point Likert scale (1 = strongly disagree to 5 = strongly agree). Psychometric evaluation demonstrated strong validity and reliability (KMO = 0.87, Bartlett’s Test *p* < 0.001, factor loadings > 0.50; Cronbach’s α = 0.89 overall, subscales 0.74–0.86). The detailed structure of the SMS-T1DC across its seven domains is presented in Table 2.

**Table 2:** Structure of the SMS-T1DC Scale (36 Items Across 7 Domains).

Domain (Abbreviation)	No. of items	Example item
Basic Diabetes Knowledge and Literacy (DBKL)	4	I know that diabetes happens because my body does not make insulin anymore.
Dietary Management (DM)	6	I know I should choose “good foods” that make my blood sugar go up slowly
Decision-Making & Problem-Solving (DMPS)	5	I know I should eat candy or a snack right away when I have low blood sugar
Insulin Administration (IA)	5	I can figure out how much insulin I need before taking it
Blood Glucose Monitoring (BGM)	6	I know when I should test my blood sugar (like before meals, after meals, and before bed)
Physical Activity (PA)	5	I know that being active and exercising is good for diabetes
Psychosocial Well-Being (PWB)	5	I try to stay in a good mood and be brave when I take care of my diabetes

Each domain is represented with one illustrative item. The full set of 36 bilingual items was administered but is not reproduced here for brevity.

##### System usability scale for children (SUS-C)

The SUS-C adapts the original SUS^44^ with simplified wording and age-appropriate anchors, following pediatric usability guidance^45^. The canonical 10 items were scored using the standard 0–100 SUS transformation; two supplementary items (“Felt Like a Superhero,” “Would Recommend to Friends”) captured narrative engagement and peer motivation and were analyzed descriptively on the 1–5 scale but not included in the composite SUS score. Reliability was good (Cronbach’s α = 0.83). Given the SUS’s widely established structural validity, and that modifications were limited to language simplification, the SUS-C is assumed to retain the canonical validity properties. A pilot confirmed comprehension and feasibility for the target age group.

##### Engagement and motivation questionnaire (EMQ)

An 8-item measure adapted from validated gameful-experience and child-motivation scales^46,47^ assessed enjoyment, willingness to continue, and perceived relevance. Reliability was good (Cronbach’s α = 0.86). Content and face validity were supported through expert review and pre-testing with a subsample of children, who confirmed clarity and age appropriateness.

### Statistical analyses

All analyses were performed in IBM SPSS Statistics (version 26). Pre–post differences in domain-level and total scores were examined using paired-sample t-tests. Effect sizes were calculated as Cohen’s d < sub > z < /sub > for paired designs (mean change divided by the standard deviation of the difference scores), with corresponding 95% confidence intervals. Assumptions were assessed through Shapiro–Wilk tests for normality of difference scores and box-plot inspection for outliers. When these assumptions were not met, Wilcoxon signed-rank tests were conducted as nonparametric sensitivity analyses, with effect sizes expressed as matched rank-biserial correlations. Statistical significance was set at *p* < 0.05 (two-tailed). Sample sizes for each analysis (N) are reported in the results tables, and all analyses were conducted on complete cases without data imputation.

### Qualitative component

In order to complement the quantitative findings, a qualitative strand provided deeper insight into user experiences within a mixed-methods design enabling triangulation^48^.

*Participants* A purposive subsample of 23 stakeholders was formally interviewed to ensure diversity of perspectives, comprising 10 children (IDs: C01–C10), 8 parents (IDs: P01–P08), and 5 healthcare providers (IDs: H01–H05, including endocrinologists, nurses, and diabetes educators). Sampling was guided by maximum variation and continued until thematic saturation was reached, at which point no substantially new themes emerged.

*Interview procedures*. Semi-structured interviews were conducted at the end of the four-week intervention, in quiet hospital playrooms or family homes to maximize comfort. Each interview lasted 20–30 min and was audio-recorded with consent. For children, age-appropriate techniques (story cards, pictorial prompts, simplified questions) reduced cognitive burden and encouraged free expression. Parents and providers were asked about observable behavioral changes, emotional responses, integration with routines, and suggestions for improvement. Observational field notes captured non-verbal behaviors (affect, collaboration styles, parental scaffolding), enriching data beyond verbal responses.

*Transcription and translation*. Recordings were transcribed verbatim in Chinese and cross-checked by bilingual research assistants. English translations were prepared, with back-translation of a random subset to verify meaning equivalence, preserving the nuances of children’s and parents’ narratives.

*Data analysis*. Thematic analysis followed Braun and Clarke’s six-step framework: (1) familiarization; (2) initial coding; (3) theme search; (4) theme review; (5) definition and naming; (6) reporting. Two independent coders analyzed transcripts in NVivo 12 (QSR International). Intercoder reliability reached 87% agreement (Cohen’s κ = 0.82, substantial). Discrepancies were resolved through discussion until consensus. Themes were triangulated across children, parents, and providers; quotations were annotated with participant IDs (e.g., C06, age 6; P11, parent of a 7-year-old; H03, nurse educator).

*Trustworthiness*. Rigor was enhanced via member checking with a subset of parents, reflexive memos documenting researcher assumptions, and triangulation across stakeholders and observations. Transparent coding and audit trails supported dependability and confirmability, aligning with best practices in HCI and pediatric health research.

## Results

### Demographics

A total of 54 children aged 4–9 years participated in the study. Table 3 presents the demographic and clinical characteristics. The sample was nearly balanced by gender (51.9% boys, 48.1% girls) and included both urban (63.0%) and rural (37.0%) households. The mean age was 6.5 ± 1.7 years, with participants evenly divided into younger (4–6 years, 48.1%) and older (7–9 years, 51.9%) subgroups to allow developmental comparison. The average duration since diagnosis was 2.3 ± 1.1 years. Prior VR exposure was limited, with 77.8% reporting no previous experience, which underscores the novelty of the intervention for most children.

**Table 3:** Demographic and clinical characteristics of participants (N = 54).

Variable	Category	n	%
Gender	Male	28	51.9
	Female	26	48.1
Residence	Urban	34	63.0
	Rural	20	37.0
Age group	4–6 years	26	48.1
	7–9 years	28	51.9
Prior VR experience	Yes	12	22.2
	No	42	77.8
Age (years)	Mean ± SD	6.5 ± 1.7	–
Duration since diagnosis (years)	Mean ± SD	2.3 ± 1.1	–

Age groups were defined a priori (4–6 vs. 7–9) to allow developmental subgroup analyses (see "Subgroup analyses by age group" section).

### Self-management outcomes (SMS-T1DC)

Paired t-test analyses revealed significant improvements across all seven domains of the SMS-T1DC (*p* < 0.001). As shown in Table 4, children demonstrated notable gains in diabetes basics, dietary management, decision-making, insulin administration, blood glucose monitoring, physical activity, and psychosocial well-being. The overall self-management score increased by more than one full scale point. All effect sizes (Cohen’s d = 0.78–1.27) were large, indicating not only statistical significance but also strong practical significance.

**Table 4:** Pre- and post-intervention outcomes by SMS-T1DC dimension (N = 54).

Outcome domain	Pre-test Mean ± SD	Post-test Mean ± SD	t-value	*p*-value	Cohen’s d
Diabetes basics	2.19 ± 0.55	3.29 ± 0.49	8.15	< 0.001	1.11
Dietary management	2.24 ± 0.60	3.28 ± 0.50	8.02	< 0.001	1.08
Decision-making & problem-solving	2.18 ± 0.58	3.25 ± 0.53	8.40	< 0.001	1.14
Insulin administration	2.09 ± 0.64	3.10 ± 0.55	7.65	< 0.001	1.03
Blood glucose monitoring	2.11 ± 0.59	3.18 ± 0.52	7.72	< 0.001	1.04
Physical activity	2.43 ± 0.63	3.70 ± 0.54	8.92	< 0.001	1.21
Psychosocial well-being	2.48 ± 0.61	3.84 ± 0.51	9.32	< 0.001	1.27
Overall self-management	2.25 ± 0.59	3.39 ± 0.51	8.95	< 0.001	1.18

Paired t-tests showed significant improvements across all domains. Effect sizes (Cohen’s d) indicate large practical gains.

*Summary* All domains improved by approximately one full Likert scale point, with large effect sizes. These quantitative gains align with qualitative reports from parents and providers, who observed greater confidence and cooperation in children’s daily diabetes care (see "Qualitative insights" section).

### Subgroup analyses by age group

To explore developmental differences, subgroup analyses compared younger (4–6 years, n = 26) and older (7–9 years, n = 28) participants, details can be seen in Table 5. Both groups demonstrated significant pre–post improvements across all domains. Effect sizes were generally larger among older children, particularly for cognitively demanding tasks such as decision-making and insulin administration. Younger children showed the strongest gains in psychosocial well-being, suggesting that narrative engagement and positive reinforcement were especially effective in reducing resistance and fostering confidence at earlier developmental stages.

**Table 5:** Subgroup analyses by age group (Cohen’s d for Pre–post changes).

Outcome domain	Ages 4–6 (n = 26)	Ages 7–9 (n = 28)
Diabetes basics	0.94	1.16
Dietary management	0.91	1.11
Decision-making & problem-solving	0.96	1.21
Insulin administration	0.88	1.12
Blood glucose monitoring	0.92	1.08
Physical activity	1.02	1.19
Psychosocial well-being	1.08	1.14
Overall self-management	1.01	1.20

Older children showed relatively greater improvements in cognitively demanding domains, while younger children benefited most in psychosocial adaptation and confidence building.

*Summary* Older children showed stronger improvements in cognitively demanding domains such as decision-making and insulin administration, whereas younger children benefited most in psychosocial well-being. These developmental patterns are consistent with interview findings that younger children engaged more deeply with the narrative for confidence-building, while older children sought greater cognitive challenge (see "Qualitative insights" section).

### Usability outcomes (SUS-C)

Children reported excellent usability for the VR serious game, with an overall mean SUS-C score of 86/100, which falls in the “excellent usability” range. More than 90% of participants were able to navigate the VR interface independently after a short introduction, and younger children (ages 4–6) required only minimal support.

As shown in Table 6, the highest endorsement was for the supplementary narrative-related item “Felt Like a Superhero” (M = 4.8, SD = 0.3), followed closely by Enjoyment (M = 4.7, SD = 0.4) and Ease of Navigation (M = 4.6, SD = 0.5). In contrast, items reflecting complexity (“unnecessarily complex” and “cumbersome to use”) received slightly lower, though still favorable, scores (> 4.0), suggesting minor opportunities for future interface streamlining.

**Table 6:** Usability questionnaire results (SUS-C, N = 54).

Item	Mean ± SD
1. I think that I would like to use this system frequently	4.5 ± 0.6
2. I found the system unnecessarily complex	4.2 ± 0.7
3. I thought the system was easy to use	4.6 ± 0.5
4. I think that I would need help to use this system	4.1 ± 0.8
5. I found the functions well integrated	4.4 ± 0.6
6. I thought there was too much inconsistency	4.3 ± 0.7
7. I imagine most children would learn to use this system quickly	4.6 ± 0.5
8. I found the system cumbersome to use	4.2 ± 0.7
9. I felt very confident using the system	4.7 ± 0.4
10. I needed to learn many things before I could use the system	4.3 ± 0.6
11. Felt Like a Superhero (supplementary)	4.8 ± 0.3
12. Would Recommend to Friends (supplementary)	4.4 ± 0.7

Scores were rated on a 5-point Likert scale (1 = strongly disagree to 5 = strongly agree). Higher scores reflect greater usability, enjoyment, and engagement. Mean values above 4.5 indicate very high acceptability of the VR intervention.

*Summary* High overall SUS-C scores confirmed the system’s accessibility and usability. Notably, the supplementary item “Felt Like a Superhero” received the highest rating, underscoring the motivational role of the narrative—a theme also emphasized by children and parents during interviews ("Qualitative insights" section).

### Engagement and motivation outcomes

Engagement and motivation outcomes were consistently high across the eight items of the Engagement and Motivation Questionnaire. As shown in Table 7, children reported strong enjoyment (M = 4.6, SD = 0.5), motivation to continue (M = 4.5, SD = 0.6), and social endorsement (“Would Recommend to Friends”, M = 4.4, SD = 0.7). These findings indicate sustained intrinsic motivation as well as peer-related reinforcement.

**Table 7:** Engagement and motivation questionnaire results (N = 54).

Item	Mean ± SD
1. I enjoyed playing the game	4.6 ± 0.5
2. I felt motivated to continue playing	4.5 ± 0.6
3. The game was interesting for me	4.6 ± 0.5
4. The game helped me learn something useful	4.4 ± 0.6
5. I want to play more sessions in the future	4.5 ± 0.6
6. The game was relevant to my life	4.3 ± 0.7
7. Willingness to Replay	4.5 ± 0.6
8. Would Recommend to Friends	4.4 ± 0.7

Scores were rated on a 5-point Likert scale (1 = strongly disagree to 5 = strongly agree). Higher scores reflect greater usability, enjoyment, and engagement. Mean values above 4.5 indicate very high acceptability of the VR intervention.

*Summary* Quantitative results revealed sustained intrinsic motivation, with high enjoyment, replay willingness, and peer endorsement. These findings were corroborated by behavioral data (high attendance, low dropout) and by qualitative feedback, in which children eagerly anticipated new sessions and parents reported extended engagement at home ("Qualitative insights" section).

Behavioral data corroborated the self-reported results. A total of 95% of children completed at least 10 of the 12 scheduled sessions, average weekly playtime was 73 min (SD = 11.2), and dropout was below 4%. Qualitative feedback from parents further highlighted the motivational pull of the narrative and achievement system, with many reporting that children spontaneously discussed characters and challenges at home, extending engagement beyond the intervention setting.

### Qualitative insights

Thematic analysis of 23 semi-structured interviews with children (C01–C10), parents (P01–P08), and healthcare providers (H01–H05) yielded four overarching themes with multiple subthemes. Data saturation was reached, as no substantially new codes emerged in the later interviews. These qualitative findings complemented the quantitative results by illustrating how the VR game influenced children’s motivation, confidence, and daily diabetes care behaviors.

### Theme 1: Strengthened confidence and reduced resistance

Children reported that the superhero narrative helped them feel “braver” when facing diabetes-related tasks, reframing procedures as playful challenges rather than sources of fear. Parents observed decreased resistance to injections and glucose checks, and healthcare providers confirmed that children appeared more willing to participate during care routines.

C06 (child, age 6): “I was not scared anymore. I felt like Superman fighting the monster.”

P05 (parent of 7-year-old): “He used to cry when it was time for injections, but now he says, ‘Let’s do it like in the game.’”

H02 (diabetes educator): “Children showed more confidence in demonstrating what they had learned.”

### Theme 2: Increased motivation and intrinsic engagement

Children expressed excitement about avatar customization, rewards, and mission-based challenges. Parents noted that this motivation carried over into daily life, with children talking about the characters and asking to replay modules at home. Providers emphasized that intrinsic curiosity was more sustained than extrinsic rewards.

C08 (child, age 7): “I liked choosing my hero clothes and fighting the monsters.”

P03 (parent of 6-year-old): “She keeps asking me, ‘When can I play again?’ even after hospital sessions.”

H04 (nurse): “Children looked forward to each session, not because they had to, but because they wanted to.”

#### Theme 3: Translation of learning into daily routines

Parents and providers reported that knowledge and skills practiced in VR were applied at home and in clinical settings. Children demonstrated greater awareness of dietary choices, recalled appropriate responses to hypoglycemia, and engaged more cooperatively in diabetes management routines.

P07 (parent of 8-year-old): “When we eat, he says, ‘This is healthy, this is not healthy,’ just like in the game.”

H03 (nurse educator): “The kids could explain hypoglycemia response better after the VR practice.”

C02 (child, age 5): “I reminded my mom, ‘Don’t forget the glucose meter!’ before meals.”

#### Theme 4: Suggestions for enhancement and future development

While the overall reception was positive, participants suggested ways to improve the intervention. Children wanted cooperative or multiplayer modes, parents requested additional educational modules (e.g., stress management, sleep hygiene), and providers recommended scaling difficulty for different age groups and including progress feedback for caregivers.

C10 (child, age 8): “I want to play with my friends, not just alone.”

P08 (parent of 6-year-old): “It would be useful to have more content about how to manage stress.”

H05 (endocrinologist): “The younger ones need simpler steps, while older children want more challenges.”

These themes and representative quotations are summarized in Table 8, which provides an overview of the key findings and illustrates how children, parents, and healthcare providers perceived the VR intervention.

**Table 8:** Themes, subthemes, and representative quotations from qualitative interviews (N = 23).

Theme	Subtheme	Stakeholder (ID)	Representative quote
Strengthened confidence & reduced resistance	Feeling braver	C06 (child, 6y)	“I was not scared anymore. I felt like Superman fighting the monster.”
	Reduced resistance to care	P05 (parent)	“He used to cry when it was time for injections, but now he says, ‘Let’s do it like in the game.’”
Increased motivation & engagement	Enjoyment of play	C08 (child, 7y)	“I liked choosing my hero clothes and fighting the monsters.”
	Sustained motivation	P03 (parent)	“She keeps asking me, ‘When can I play again?’ even after hospital sessions.”
Translation into daily routines	Dietary choices	P07 (parent)	“When we eat, he says, ‘This is healthy, this is not healthy,’ just like in the game.”
	Emergency readiness	H03 (nurse educator)	“The kids could explain hypoglycemia response better after the VR practice.”
Suggestions for enhancement	Peer interaction	C10 (child, 8y)	“I want to play with my friends, not just alone.”
	Developmental scaling	H05 (endocrinologist)	“The younger ones need simpler steps, while older children want more challenges.”

Quotations were translated from Chinese and lightly edited for clarity while preserving participants’ intended meaning. Themes and subthemes align with the CDS-7 domains of self-management where relevant.

*Summary* Taken together, the four qualitative themes—strengthened confidence, increased motivation, translation of learning into routines, and suggestions for enhancement—complement the quantitative outcomes. They illustrate how improvements in CDS-7 domains were experienced by children and families in daily life, highlighting the added value of narrative-driven VR in pediatric diabetes self-management.

## Discussion

### Key findings

This study demonstrates that a narrative-driven VR serious game, designed through a child-centered participatory process, can significantly improve pediatric type 1 diabetes (T1D) self-management. Statistically significant gains were observed across all seven CDS-7 domains, with large effect sizes, confirming the educational and motivational potential of immersive gameplay. Improvements were most pronounced in domains requiring experiential learning—such as dietary management, decision-making, and psychosocial well-being—underscoring VR’s capacity to translate abstract medical concepts into tangible, engaging experiences.

The subgroup analysis revealed developmental differences: older children (7–9 years) showed stronger gains in cognitively demanding tasks (e.g., insulin administration, decision-making), while younger children (4–6 years) particularly benefited in psychosocial adaptation and confidence. This aligns with developmental theory, suggesting that younger children rely more on metaphor and narrative role-play to overcome fear, whereas older children are increasingly capable of abstract reasoning and procedural mastery.

Qualitative findings provided explanatory depth. Parents and healthcare providers described reductions in children’s resistance to injections and glucose checks, while children expressed excitement, pride, and a sense of agency. These converging results indicate that the VR game served not merely as an instructional tool but as a motivational scaffold, reshaping children’s affective orientation toward diabetes self-care.

### Contributions to HCI and digital health

This study advances HCI and pediatric digital health research in three key ways:

#### Participatory design with young children

The study demonstrates the feasibility of engaging children under 7 years of age as active co-designers, using workshops, storyboarding, and iterative playtesting. Such involvement ensured developmental appropriateness and emotional safety, representing a significant departure from the clinician-driven approaches that dominate pediatric digital health design.

#### Operationalization of CDS-7 in VR Mechanics

To our knowledge, this is among the first studies to systematically map the CDS-7 self-management framework into VR game modules. By aligning each domain with tailored mechanics, the intervention maintained educational fidelity while enhancing experiential engagement. For example, transforming hypoglycemia response into an interactive branching “adventure” enabled both procedural rehearsal and emotional desensitization.

#### Mixed-methods evaluation framework

The combination of validated psychometric tools, adherence metrics, and thematic qualitative analysis provides a replicable framework for evaluating pediatric digital interventions. This triangulation moves beyond short-term knowledge testing, offering a multidimensional view of usability, engagement, and psychosocial impact.

Together, these contributions illustrate a new paradigm for pediatric digital health: interventions that are not only evidence-based and clinically accurate but also developmentally resonant and experientially meaningful.

### Limitations and future directions

This study has several limitations that must be acknowledged. First, the single-group pre–post design precludes strong causal inference, as improvements could in part reflect maturation, testing, or novelty effects. Second, the four-week intervention period limits conclusions about long-term sustainability; future studies should extend to 6–12 month follow-up to evaluate maintenance of effects. Third, outcomes relied primarily on validated self-report questionnaires rather than biomedical endpoints such as HbA1c or continuous glucose monitoring, reducing clinical generalizability. Fourth, the modest sample size (N = 54) constrains statistical power, and social desirability bias may have influenced parent- and child-reported outcomes. Fifth, the study was conducted in a single regional context in China, which may limit cross-cultural applicability; narrative elements such as the “superhero” metaphor may require localization in other cultural settings. Finally, the absence of an active comparator group—such as traditional education or existing mobile-based serious games—limits the ability to determine the incremental advantage of immersive VR over other educational modalities.

To address these limitations, future studies should employ multi-arm randomized controlled trials with active comparators (e.g., traditional teaching, mobile applications) to establish incremental efficacy. Longitudinal follow-up (6–12 months) and integration of biomedical endpoints (HbA1c, CGM data) will be essential to evaluate sustained clinical benefits. Expanding recruitment to multiple centers and diverse cultural contexts will enhance generalizability and allow testing of culturally localized narratives. Finally, incorporating parental dashboards, adaptive difficulty, and dosage guidelines may optimize safe and scalable implementation in real-world pediatric care.

### Implications for practice

The findings hold practical implications for healthcare, design, and research practice:

*For healthcare providers*: The VR platform can serve as a complementary tool to traditional diabetes education, especially in reducing fear and resistance to routine tasks. It offers a safe space for rehearsal, potentially improving adherence in real-world care.

*For designers and researchers*: Multidisciplinary collaboration—including endocrinologists, psychologists, educators, and parents—proved essential for ensuring clinical accuracy, developmental fit, and emotional safety. Such collaboration should be regarded as a prerequisite rather than an optional enhancement.

*For future development*: Design refinements should prioritize adaptive difficulty scaling, cross-cultural narrative localization, and caregiver-facing dashboards to balance engagement with safety.

This research provides preliminary but compelling evidence that a child-centered, narrative-driven VR serious game can positively influence multiple dimensions of pediatric T1D self-management. By embedding theoretical rigor within immersive storytelling and grounding development in participatory HCI methods, the study advances a replicable model for digital health innovation. Future research should validate these findings through controlled trials, biomedical endpoints, multi-center studies, and long-term follow-up. If confirmed, narrative-driven VR interventions may become a powerful adjunct to conventional care, empowering children and families to engage more confidently and sustainably in diabetes self-management.

## Conclusion

This project set out to design, develop, and evaluate *Tangbao Superman Transformation*, a narrative-driven virtual reality (VR) serious game for pediatric type 1 diabetes (T1D) self-management. Guided by Human–Computer Interaction (HCI) principles, the study integrated the Chinese Diabetes Society’s seven-domain model (CDS-7) with participatory design, usability testing, and empirical evaluation.

The research makes three overarching contributions. First, it advances theory by demonstrating how established diabetes self-management frameworks can be operationalized in immersive VR mechanics, thereby bridging medical models with experiential learning. Second, it contributes methodologically by applying participatory design with very young children (ages 4–9), illustrating both feasibility and value in capturing authentic developmental perspectives that are often excluded in digital health design. Third, it contributes practically by delivering empirical evidence that child-centered VR interventions can enhance self-management knowledge, reduce resistance, and sustain motivation in real-world pediatric contexts.

Beyond its immediate findings, the study highlights the broader role of narrative-driven VR in reimagining pediatric digital health interventions. By transforming potentially intimidating medical routines into playful and empowering experiences, VR can function as both an educational scaffold and a motivational catalyst. This dual role—linking clinical accuracy with affective engagement—offers a replicable model for designing digital health tools that are developmentally appropriate, culturally sensitive, and ethically sound.

Nonetheless, the research remains preliminary. Limitations related to sample size, homogeneity, short intervention duration, reliance on self-reported outcomes, and the absence of biomedical indicators must be acknowledged. These constraints underscore the need for future randomized controlled trials, longitudinal follow-up, integration of clinical biomarkers, and validation across diverse cultural and healthcare contexts.

In conclusion, this work contributes to the emerging field of child-centered digital health by providing a theoretically grounded, empirically tested, and ethically robust model for VR-based diabetes self-management. It demonstrates that narrative-driven, participatory HCI approaches can not only improve children’s immediate learning and confidence but also lay the groundwork for scalable and sustainable innovations in chronic disease management. With continued refinement and validation, such interventions hold the potential to transform pediatric healthcare by empowering children and families to take an active, confident, and motivated role in managing lifelong conditions.

## Data Availability

All data generated or analyzed during this study are included in this article. Additional information can be obtained from the corresponding author upon reasonable request.
